# The Usability of a Pressure-Indicating Film to Measure the Teat Load Caused by a Collapsing Liner

**DOI:** 10.3390/s16101597

**Published:** 2016-09-28

**Authors:** Susanne Demba, Sabrina Elsholz, Christian Ammon, Sandra Rose-Meierhöfer

**Affiliations:** 1 Leibniz Institute for Agricultural Engineering and Bioeconomy e.V. (ATB), Department of Engineering for Livestock Management, Max-Eyth-Allee 100, Potsdam 14469, Germany; selsholz@atb-potsdam.de (S.E.); cammon@atb-potsdam.de (C.A.); 2 Hochschule Neubrandenburg, University of Applied Sciences, Department of Agricultural Machinery, Brodaer Straße 2, Neubrandenburg 17033, Germany; rose@hs-nb.de

**Keywords:** teat load, liner collapse, pressure-indicating film, artificial teat, machine milking

## Abstract

Prevention of damage to the teat and mastitis requires determination of the teat load caused by a collapsing liner. The aim of this study was to test a pressure-indicating film designed to measure the pressure between a collapsing liner and artificial teats. The Ultra Super Low and the Extreme Low pressure-indicating films were tested on two types of artificial teat. The experiments were performed with a conventional milking cluster equipped with round silicone liners. For each teat and film type, 30 repetitions were performed. Each repetition was performed with a new piece of film. Kruskal-Wallis tests were performed to detect differences between the pressure values for the different teats. The area of regions where pressure-indication color developed was calculated to determine the most suitable film type. Both film types measured the pressure applied to both artificial teats by the teat cup liner. Thus, the pressure-indicating films can be used to measure the pressure between a collapsing liner and an artificial teat. Based on the results of the present investigation, a pressure-indicating film with the measurement ranges of both film types combined would be an optimal tool to measure the overall pressure between an artificial teat and a collapsing liner.

## 1. Introduction

The teat cup liner is the interface between the teat of a dairy cow and the milking machine. A milking system that works improperly can damage the teat and can increase the risk of udder infections. To evaluate the influence of machine milking on the teat condition, various scoring systems evaluating teat color, swellings, ring formation at the teat base, and hyperkeratosis are used [1,2]. However, these methods are subjective. Therefore, methods to detect the pressure between the teat and a collapsing liner have been developed. One such method frequently used in literature is the calculation of the touch point [3,4,5,6,7,8], the residual vacuum available for massage [4,9,10], the liner compression [3,4,8,9], the over-pressure [4,10,11], and the true milk:rest ratio [4,10]. In several studies, the usability of pressure sensors to measure the pressure between a teat and the teat cup liner was tested. Muthukumarappan et al. [12] tested the usability of thin-film force sensors to measure the compressive load applied to the teat by different teat cup liners. The authors found both sensors unsatisfactory. One sensor showed significant error caused by bending and the other sensor had insufficient sensitivity. Adley and Butler [13] used a load cell-containing aluminum teat to measure the forces applied by a teat cup liner. The teat they used consisted of three hollow cylindrical sections. One of these sections had a radial hole in which a piston was fitted. The inner end of this piston was in contact with a miniature load cell inside the teat when pressure was applied. The other cylindrical section could be removed to shorten the teat or to interchange the sections to change the position of the load cell. Davis et al. [14] developed a device similar to the teat sensor used by Adley and Butler [13], but their device deformed much like live teats. Therefore, they mounted a miniature load cell on a steel plate. The sensing surface was thus flush with the surface of the steel plate. The sensor was covered using natural gum rubber and a gel-like material. Tol et al. [15] investigated the teat-liner interface with the help of a flexible pressure-sensitive layer. The layer included carbon particles and was attached to a resin film through which electrodes faced each other. If pressure was applied to the resin film, the distance between the carbon particles was reduced, the tunneling phenomenon occurred and the electrical resistance between the electrodes decreased. Leonardi et al. [16] used an artificial teat sensor adapted from Davis, Reinemann and Mein [14]. The authors mounted their force sensor on a flat plastic plate with a 9.5-mm-radius rounded end as the active area of the force sensor. The end of the sensor was covered by a cylinder with an end molded from silicone (Shore A hardness 10). According to the authors, this sensor is useful only for round liners.

The Prescale pressure-indicating film by Fujifilm (KAGER Industrieprodukte GmbH, Dietzenbach, Germany) is used to measure pressure, pressure distribution, and pressure balance. The Prescale pressure-indicating films are available in mono- and two-sheet types. The mono-sheet type has a polyester base containing the color-developing materials. In contrast, the two-sheet film consists of a color-forming layer and a color-developing layer. When pressure is applied to the Prescale film, the microcapsules are broken, and the color-forming material reacts with the color-developing material. As a result, red patches appear on the film, and the density of the red color indicates several levels of pressure. The Prescale pressure-indicating films measure pressures between 0.05 MPa and 300 MPa [17]. They do not support shear stress [18], and shear stress can alter the color intensity measured by the film [19].

As the methods commonly used to detect the teat load caused by the liner use indirect estimation, and as the tested sensors have shown limited usability, the aim of this study was to determine whether the Prescale pressure-indicating films developed by Fujifilm can be used to measure the teat load caused by liner collapse.

## 2. Materials and Methods

### 2.1. Study Design and Data Collection

The Ultra Super Low (Film 1) and the Extreme Low (Film 2) films (Prescale by Fujifilm, KAGER Industrieprodukte GmbH) were tested. Both are two-sheet film types with a thickness of 0.1 mm, and both consist of an A-film (Side A) and a C-film (Side C). Side A is composed of a polyester base and a microencapsulated color-forming layer. The components of Side C are a color-developing layer and a polyester base. To measure an applied pressure, both sides must adjoin each other at the rough sides. If the sides do not adjoin each other while a pressure is applied, no reaction and therefore no color change takes place. The pressure ranges are 0.2–0.6 MPa for Film 1 and 0.05–0.2 MPa for Film 2.

**Figure 1:**
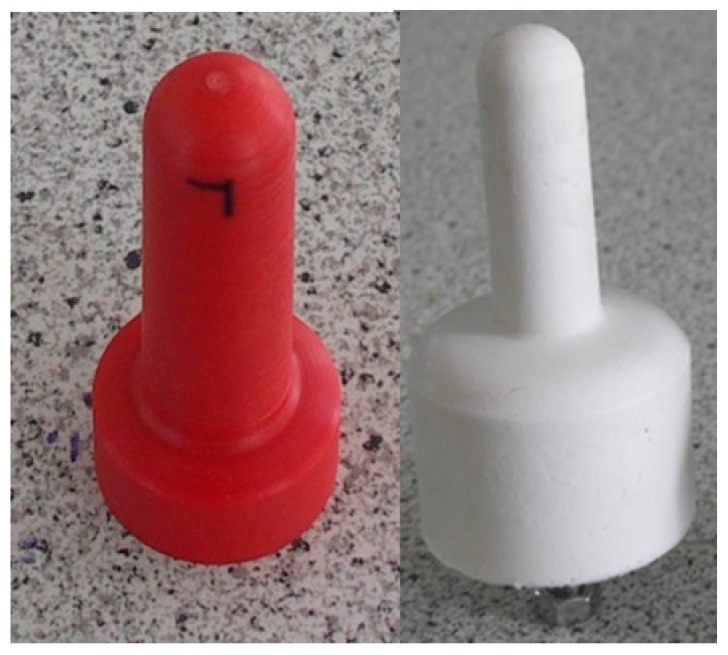
The plastic teat (**left**) and the silicone teat (**right**) used in this investigation.

Two experiments were conducted in this investigation. To test whether the two pressure-indicating films measured the load of the liner in the teat cup during liner collapse, two types of pressure-indicating film were tested on an artificial, stiff plastic teat in the first experiment (E1). The plastic teat was 54 mm in length with an average diameter of 21.5 mm. To test whether the pressure-indicating films measured the pressure between two flexible objects, both types of pressure-indicating film were tested on a flexible, silicone rubber teat in the second experiment (E2). This teat had a length and a mean diameter of 56 mm and 21 mm, respectively. According to the manufacturer, the silicone rubber had a Shore A hardness 25, a density of 1.16 g∙cm^−3^ at a temperature of 23 °C, a tensile strength of 5.00 N∙mm^−2^, an ultimate elongation of 350%, a tear resistance of more than 20 N∙mm^−1^, and a linear shrinkage of 0.5%. The plastic and silicone teats are shown in Figure 1.

**Figure 2:**
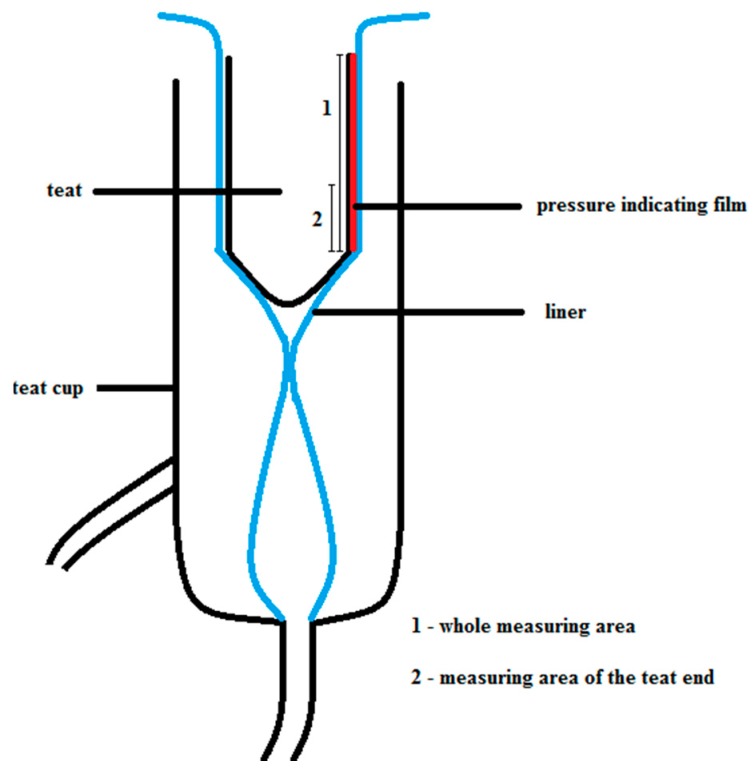
Schematic drawing of the position of the teat equipped with the pressure-indicating film.

All experiments were performed in the laboratory milking parlor of the Leibniz Institute for Agricultural Engineering and Bioeconomy. A conventional milking cluster equipped with round silicone liners was used to measure the teat load caused by the liner. The milking cluster consisted of four teat cups, each with a short milk tube and a short pulse tube. The short milk tubes joined in a claw where the milk of the whole udder normally flows together. The IQPro liner by GEA (GEA Group Aktiengesellschaft, Düsseldorf, Germany) was used in this investigation. The liner had a shaft diameter of 24 mm, a mouthpiece diameter of 21 mm, and a head diameter of 58 mm. All measurements were taken in the same teat cup. In order to test whether the measurement values are repeatable the measurements were carried out under constant conditions. Therefore, the machine vacuum was adjusted at 40 kPa. A pulsation rate of 60 min^−1^ and a pulsation ratio of 60:40 were used. The pressure-indicating film was cut into 35 mm × 45 mm pieces, and the pieces were attached at the teat with tape. To ensure that all film pieces were attached in the same position, the teats were marked (Figure 1). The teat with the pressure-indicating film was inserted into the teat cup, and the liner was opened and closed for 30 s so that the liner collapsed 30 times. The teat was aligned in the teat cup with the collapsed liner, and the sides of the pressure-indicating film were pressed together (Figure 2). For both types of teat and film, 30 repetitions were performed. Each repetition was done with a new piece of film. There was no milk or water flow involved in the experiments because the artificial teats were not hollow and thus not suitable to involve milk or water flow.

After the measuring procedure, the C-sides of the films were visualized with a scanner (Epson Perfection V37/V370 Photo, KAGER Industrieprodukte GmbH) and analyzed with the FPD-8010E software by Fujifilm (KAGER Industrieprodukte GmbH). This software was used to analyze the six parameters proportionately within the film’s pressure-detection range (effective rate, ER, in %), the surface area on which the color was generated (pressed area, PA, in mm^2^), the mean pressure on the area on which the color was generated (average pressure, AP, in MPa), the maximum pressure of the area on which the color was generated (maximum pressure, MP, in MPa), the product of the pressurization surface area and average pressure (load, L, in N), and the measured area (MA, in mm^2^). In this investigation, the values of AP, MP, and the colored area (CA) were used to evaluate the usability of the Prescale pressure-indicating film to measure the teat load caused by a collapsing liner. CA was calculated as follows:

CA = PA/MA(1)

where CA is colored area in %; PA is pressed area in mm^2^; and MA is measured area in mm^2^.

AP, MP, and CA were calculated for the whole area of the film piece as well as for the area of the teat end. The area of the teat end was defined as the area of the lower third of the barrel of the artificial teats. These calculations were performed for each film and teat.

### 2.2. Pretest: Influence of Bending and Negative Pressure on the Measurements

To investigate the influence of bending on the pressure-indicating film, the films were attached at the teats for 2 min. Two attachment methods were tested. The pressure-indicating films were directly attached with Side A as well as with Side C on the teat. For each teat, film type, and attachment method five repetitions were done, each repetition with a new piece of film. AP, MP, and CA were calculated.

To analyze the influence of negative pressure on the measuring results, a mobile milker (Minimelker, schlauerbauer Melktechnik GmbH, Leipzig, Germany) was used. The pressure-indicating film was attached inside at the bottom of the milk can of the mobile milker and the teat cups were closed with the help of plugs. The machine pressure was adjusted at −41 kPa and the film was exposed to the negative pressure for 30 s. For each film type five repetitions were done, each repetition with a new film piece. AP, MP, and CA were analyzed.

### 2.3. Statistical Analyses

Data were analyzed with the SAS software package 9.4 (SAS Institute Inc., Cary, NC, USA). The UNIVARIATE procedure was used to calculate descriptive statistics for AP and MP. Kruskal-Wallis-tests were performed to estimate differences between the two artificial teats and between areas regarding AP and MP using the NPAR1WAY procedure because the distribution of the measurement values of both traits was not symmetrical. The null hypothesis was that there were no differences between the teats and the areas.

The GLIMMIX procedure was used for a one-way ANOVA to examine the percentage of CA for each teat and film type. As the observations were percentage values, a binomial distribution with a logit link function was used. The linear predictor η is calculated as follows:

η_i_ = µ + F_i_ + ε_i_ (2)

where µ is the general mean; F_i_ is the fixed effect of film i (Film 1, Film 2); and ε_i_ is the residual. The null hypothesis was that there were no differences between the tested films regarding the color-developing area.

To analyze the influence of bending and negative pressure on the pressure values measured by the pressure-indicating film, descriptive statistics for AP and MP were calculated using the UNIVARIATE procedure. The GLIMMIX procedure was used to examine the percentage of CA for each teat and film type. A t-test was carried out to investigate differences between the attachment methods of the films. The null hypothesis was that there were no differences between the methods. All tests were carried out at a significance level of 0.05.

## 3. Results

### 3.1. Influence of Liner Collapse on Teat Load

The median, minimum, 25% quantile, 75% quantile, and maximum values of AP and MP for both teats, tested areas, and film types are given in detail in Table 1.

**Figure 3:**
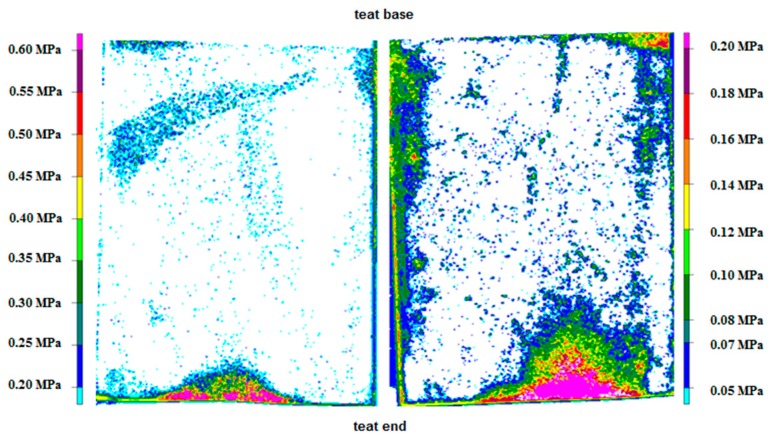
Pressure levels and pressure distribution on a plastic teat caused by the collapsing liner measured with Film 1 (**left**) and Film 2 (**right**).

In E1, both film types measured the pressure and the pressure distribution between the teat cup liner and the plastic teat. For Film 1, AP and MP were 0.22 MPa and 0.63 MPa for the whole film piece and 0.26 MPa and 0.63 MPa for the teat end, respectively. The pressure values of Film 2 were between 0.08 MPa and 0.23 MPa for the whole film piece and between 0.09 MPa and 0.21 MPa for the teat end. Figure 3 shows an example of the pressure level and the pressure distribution for Film 1 and Film 2.

**Figure 4:**
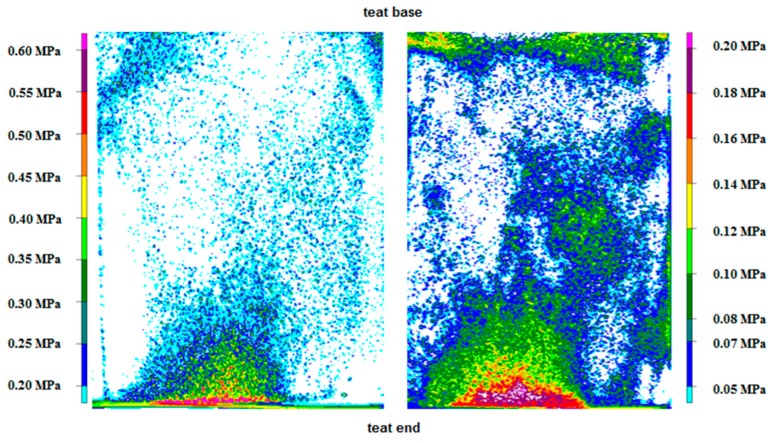
Pressure levels and pressure distribution on a silicone teat caused by the collapsing liner measured with Film 1 (**left**) and Film 2 (**right**).

In E2, Film 1 and Film 2 measured the pressure and the pressure distribution between the liner and a silicone teat (Figure 4). The pressure values of Film 1 and the whole film piece were 0.22 MPa and 0.62 MPa for AP and MP, respectively. For the teat end, AP and MP were 0.25 MPa and 0.61 MPa for Film 1, respectively. For Film 2, the measurements were 0.09 MPa (AP) and 0.21 MPa (MP) for the whole film piece, as well as for the teat end.

The results of the Kruskal-Wallis tests show significant differences between teats regarding the values of AP and MP. These differences depend on the measuring area and the film type. The pressure values of Film 1 show significant differences between both artificial teats for AP of the whole area (*p* = 0.0397) and for MP of the teat end area (*p* = 0.023). The pressure values of MP of the teat end were higher for the plastic teat. Regarding AP of the whole area, the pressure was higher on the teat made of silicone. In comparison, with Film 2, the pressure values of the plastic teat were higher than those of the silicone teat for MP of the whole area (*p* < 0.0001), as well as for AP (*p* = 0.0005) and MP (*p* < 0.0001) of the teat end area. The values of AP of the whole area were higher for the silicone teat (*p* = 0.0001).

Significant differences between measuring areas could be found as well. The values of AP of both Film 1 (*p* < 0.0001; *p* < 0.0001) and Film 2 (*p* < 0.0001; *p* = 0.003) were higher in the teat end area than in the whole area for both artificial teats (plastic and silicone, respectively). No significant differences could be found for MP.

The differences in the color-developing area between teats and film types are given in Table 2.

With the plastic teat, on Film 1, color developed on 13.5% of the whole area and on 18.8% of the teat end area. The color-developing area of Film 2 was 36.7% of the whole area and 46.4% of the teat end area.

On the silicone teat, the color-developing area was 22% of the whole area on Film 1 and 75% on Film 2. On the teat end area, color developed on 24% of Film 1 and on 88% of Film 2. Thus, more color developed on Film 2 and in the teat end area, indicating that more pressure was applied on the teat end.

### 3.2. Pretest: Influence of Bending and Negative Pressure on the Measurements

Table 3 shows the influence of bending on the pressure-indicating film by artificial teat, attachment method, and film type.

Depending on artificial teat and film type, CA ranged between 1.1% and 10.4% when Side A was attached directly to the teat. The pressure values were between 0.06–0.17 MPa for AP and 0.14–0.37 MPa for MP. When Side C was attached directly to the teat, CA was between 3.2% and 10.8%. With this attachment method, the measured pressure for AP and MP was 0.06–0.18 MPa and 0.14–0.44 MPa, respectively. No significant differences between the attachment methods and their influence on the measurement results were found by the t-test.

The results regarding the influence of negative pressure on the measurements of the pressure-indicating film show that on Film 1, color developed on 0.03% of the film area. The pressure values of Film 1 for AP and MP were 0.16 MPa and 0.22 MPa, respectively. On Film 2, color developed on 0.01% of the film area. The measured values were 0.05 MPa for AP and 0.09 MPa for MP.

## 4. Discussion

Depending on artificial teat, film type, and measuring area, the pressure applied due to a collapsing liner ranged between 0.07 MPa (70 kPa) and 0.64 MPa (640 kPa). These pressure values are much higher than those found in other investigations. The pressure measured by Tol, Schrader and Aernouts [15] was 99–180 kPa at the teat end. Muthukumarappan et al. [20] measured a pressure of 18–35 kPa between a teat and a collapsing liner. Depending on the vacuum in the short milk tube and the liner design, Leonardi, Penry, Tangorra, Thompson and Reinemann [16] found pressure values between 20 kPa and 34 kPa. Davis, Reinemann and Mein [14] detected pressures of 20–41 kPa between the liner and teat. The artificial teats used in the present investigation could explain why the pressure values measured in this investigation are higher: in the other investigations the artificial teats were hollow and made of silicone [15]; our artificial teats were not hollow. However, the silicone teat used in the present investigation was very stiff. In future investigations, a hollow silicone teat will be used to detect the pressure caused by a collapsing liner. Differences in material offer an additional explanation. Adley and Butler [13] used a teat made of aluminum and the artificial teat of Muthukumarappan, Reinemann and Mein [12] was liquid-filled, flexible, not extensible and made of a plastic teat cup plug, a surgical glove finger, and a cloth glove finger. The artificial teat of Davis, Reinemann and Mein [14] contained natural gum rubber or a gel-like material. Leonardi, Penry, Tangorra, Thompson and Reinemann [16] used a silicone rubber with a Shore A hardness of 10. In the present investigation, a silicone rubber with a Shore A hardness of 25 was used.

With both tested teats, more pressure was found on the teat end compared to the whole teat. These results agree with those of Tol, Schrader and Aernouts [15]. They had found that the maximum pressure was always exerted on the teat end. The study of Muthukumarappan, Reinemann and Mein [12] showed that the maximum pressure was applied within 1 or 2 mm of the teat end. They detected a progressive decrease in the applied pressure over the upper 3 or 4 mm of the teat end.

Film 2 was more suitable as a tool to measure the pressure between a collapsing liner and both artificial teats than Film 1 was because more color developed on Film 2. The specific pressure range of Film 2 (0.05–0.2 MPa) could explain this finding. The analyses of the film types showed white pixels in the area of the teat end, possibly as a result of the pressure range of the film types. The pressure seems to be higher or lower at these points than the films can measure. Thus, the pressure ranges of neither film alone were sufficient to measure the overall load between the collapsed liner and the artificial teats. Therefore, a pressure-indicating film with the pressure range of both types of film would be a useful tool to measure the pressure between these artificial teats and the liner.

The influence on the measurement results of bending and negative pressure on the pressure-indicating film was not significant.

## 5. Conclusions

In general, both Film 1 and Film 2 measured the pressure and the pressure distribution between either a plastic teat or a silicone teat and a collapsing liner. The pressure-indicating film is not influenced by bending or negative pressure. It can be used to detect differences between teats regarding the applied pressure as well. Thus, the Prescale pressure-indicating film can be used to measure the pressure between a collapsing liner and the artificial teats. Based on the results of this investigation, a pressure-indicating film that includes the measured ranges of both film types (0.05–0.6 MPa) would be useful. However, the usefulness of the pressure-indicating films to measure the pressure between two flexible objects remains to be determined, and, therefore, further studies are required. The influence of milking settings and different liner types on the teat load will be examined as well.

## Figures and Tables

**Table 1:** Median, minimum, 25% quantile (Q1), 75% quantile (Q3), and maximum values of average pressure (AP) and maximum pressure (MP) of both teats, tested areas, and film types.

Teat	Area	Film-Type	Variable	Median	Minimum	Q1	Q3	Maximum
Plastic	Whole	Film 1	AP	0.22	0.20	0.21	0.22	0.23
			MP	0.64	0.47	0.64	0.64	0.64
		Film 2	AP	0.08	0.07	0.07	0.08	0.08
			MP	0.23	0.21	0.23	0.23	0.24
	Teat end	Film 1	AP	0.26	0.22	0.25	0.27	0.33
			MP	0.64	0.46	0.64	0.64	0.64
		Film 2	AP	0.10	0.09	0.10	0.11	0.11
			MP	0.23	0.21	0.23	0.23	0.24
Silicone	Whole	Film 1	AP	0.22	0.20	0.22	0.23	0.27
			MP	0.64	0.52	0.64	0.64	0.64
		Film 2	AP	0.08	0.08	0.08	0.09	0.10
			MP	0.21	0.19	0.20	0.22	0.23
	Teat end	Film 1	AP	0.25	0.21	0.23	0.27	0.29
			MP	0.64	0.44	0.58	0.64	0.64
		Film 2	AP	0.09	0.08	0.09	0.10	0.12
			MP	0.21	0.19	0.20	0.22	0.23

**Table 2:** Mean and lower and upper 95% confidence interval (CI) for the mean of the colored area (%) of all tested teats, film types, and areas.

Teat	Film Type	Area	Mean	Lower 95% CI of Mean	Upper 95% CI of Mean
Plastic	Film 1	Whole	13.5	5.1	31.2
		Teat end	18.8	8.3	37.1
	Film 2	Whole	36.7	21.4	55.3
		Teat end	46.4	29.3	64.3
Silicone	Film 1	Whole	21.7	10.3	40.2
		Teat end	24.0	11.8	42.6
	Film 2	Whole	75.1	56.4	87.2
		Teat end	88.3	70.7	95.9

**Table 3:** Colored area (CA), average pressure (AP), and maximum pressure (MP) with 95% confidence intervals (CI) by artificial teat, side of attachment (Side A or Side C directly on the teat), and film type after bending of the pressure-indicating films.

Teat	Side	Film Type	CA (%)	CI of the Mean		AP (MPa)	CI		MP (MPa)	CI	
				Lower	Upper		Lower	Upper		Lower	Upper
Plastic	A	Film 1	1.1	0.000	99.5	0.17	0.16	0.17	0.35	0.30	0.41
		Film 2	9.9	0.35	77.6	0.06	0.06	0.07	0.15	0.13	0.17
Silicone	A	Film 1	3.4	0.01	91.3	0.17	0.17	0.18	0.37	0.32	0.45
		Film 2	10.4	0.39	77.3	0.06	0.06	0.06	0.14	0.11	0.15
Plastic	C	Film 1	3.8	0.02	89.8	0.18	0.17	0.18	0.42	0.39	0.44
		Film 2	10.8	0.43	77.1	0.06	0.06	0.07	0.16	0.14	0.23
Silicone	C	Film 1	3.2	0.009	92.2	0.18	0.17	0.19	0.44	0.34	0.55
		Film 2	4.2	0.003	88.2	0.06	0.06	0.07	0.14	0.12	0.16

## Abbreviations

AP	average pressure
CA	colored area
CI	confidence interval
E1	experiment one
E2	experiment two
ER	effective rate
Film 1	Ultra Super Low Prescale pressure-indicating film
Film 2	Extreme Low Prescale pressure-indicating film
L	load
MA	measured area
MP	maximum pressure
PA	pressed area
Q1	25% quantile
Q3	75% quantile
Side A	the A-film of the pressure-indicating film
Side C	the C-film of the pressure-indicating film
STD	standard deviation

## References

[1] Neijenhuis F., Barkema H.W., Hogeveen H., Noordhuizen J.P.T.M. (2000). Classification and longitudinal examination of callused teat ends in dairy cows. J. Dairy Sci..

[2] Mein G.A., Neijenhuis F., Morgan W.F., Reinemann D.J., Hillerton J.E., Baines J.R., Ohnstad I., Rasmussen M.D., Timms L., Britt J.S. Evaluation of bovine teat condition in commercial dairy herds: 1. Non-infectious factors. Proceedings of the International Symposium on Mastitis and Milk Quality.

[3] Alejandro M., Roca A., Romero G., Diaz J.R. (2014). Effects of overmilking and liner type and characteristics on teat tissue in small ruminants. J. Dairy Res..

[4] Mein G.A., Reinemann D.J. Biomechanics of milking: Teat-liner interaction. Proceedings of the American Society of Agricultural and Biological Engineers.

[5] Rosca R., Carlescu P., Tenu I., Chirila C. (2012). Evaluation of a data aquision system for measuring the milking machine process parameters. Sci. Pap. Anim. Sci. Ser..

[6] Spencer S.B., Jones L.R. (2000). Liner wall movement and vacuum measured by data acquisition. J. Dairy Sci..

[7] Spencer S.B., Shin J.W., Rogers G.W., Cooper J.B. (2007). Short communication: Effect of vacuum and ratio on the performance of a monoblock silicone milking liner. J. Dairy Sci..

[8] Zucali M., Reinemann J.D., Tamburini A., Bade R.D. Effects of liner compression on teat-end hyperkeratosis. Proceedings of the American Society of Agricultural and Biological Engineers.

[9] Bade R.D., Reinemann D.J., Zucali M., Ruegg P.L., Thompson P.D. (2009). Interactions of vacuum, b-phase duration, and liner compression on milk flow rates in dairy cows. J. Dairy Sci..

[10] Mein G.A., Williams D.M.D., Reinemann D.J. Effects of milking on teat-end hyperkeratosis: 1. Mechanical forces applied by the teatcup liner and responses of the teat. Proceedings of the 42nd Annual Meeting of the National Mastitis Council.

[11] Neijenhuis F., Klungel G.H., Hogeveen H., Noordhuizen J.P.T.M. (2005). Machine Milking Risk Factors for Teat End Callosity in Dairy Cows on Herd Level.

[12] Muthukumarappan K., Reinemann D.J., Mein G.A. Compressive load applied by the teatcup liner to the bovine teat. Proceedings of the American Society of Agricultural Engineers Meeting.

[13] Adley N.J.D., Butler M.C. (1994). Evaluation of the use of an artificial teat to measure the forces applied by a milking machine teatcup liner. J. Dairy Res..

[14] Davis M.A., Reinemann D.J., Mein G.A. Development and testing of a device to measure compressive teat load applied to a bovine teat by the closed teatcup liner. Proceedings of the American Society of Agricultural and Biological Engineers Annual Meeting.

[15] Van de Tol P.P.J., Schrader W., Aernouts B. (2010). Pressure distribution at the teat-liner and teat-calf interfaces. J. Dairy Sci..

[16] Leonardi S., Penry J.F., Tangorra F.M., Thompson P.D., Reinemann D.J. (2015). Methods of estimating liner compression. J. Dairy Sci..

[17] Cuffaro V. (2013). Prediction Method for the Surface Damage in Splined Couplings. Ph.D. Thesis.

[18] Rodríguez-Martínez R., Urriolagoitia-Sosa G., Torres-San Miguel C.R., Hernández-Gómez L.H., Urriolagoitia-Calderón G., Carbajal-Romero M.F. (2012). Development of an experimental apparatus for testing a total knee prostheses focused on mexican phenotype. Int. J. Phys. Sci..

[19] Patterson R., Pogue D., Viegas S. (1997). The effects of time and light exposure on contact and pressure measurements using Fuji prescale film. Iowa Orthop. J..

[20] Muthukumarappan K., Reinemann D.J., Mein G.A. Compressive load applied to the bovine teat by the teatcup liner. Proceedings of the American Society of Agricultural Engineers International Winter Meeting.

